# The Congenital Malformation of the Interatrial Septum—A Review of Its Development and Embryology with Clinical Implications

**DOI:** 10.3390/jdb13030028

**Published:** 2025-08-05

**Authors:** Rui Caetano Oliveira, Paula Martins, Maria de Fátima Martins

**Affiliations:** 1 Instituto de Histologia e Embriologia, Faculdade de Medicina da Universidade de Coimbra, 3000-370 Coimbra, Portugal; 2 Centro de Anatomia Patológica Germano de Sousa, 3030-075 Coimbra, Portugal; 3 Centro de Investigação em Meio Ambiente, Genética e Oncobiologia (CIMAGO), 3001-301 Coimbra, Portugal; 4 Unidade Local de Saúde, 3000-075 Coimbra, Portugal

**Keywords:** heart, human development, atrial septal defects

## Abstract

The development process of the heart and cardiovascular system is fundamental in human development and highly regulated by genetic factors. This process needs to be highly regulated to prevent malformations. Nevertheless, some heart defects may be identified, especially with modern imaging methodology. Atrial septal defects (ASDs) are particularly common. Understanding the mechanisms involved in ASD formation is fundamental for developing new treatment strategies. In this article, we explore cardiac development and embryology, with a focus on atrial septal defects and their clinical implications.

## 1. Introduction

The development of the heart and cardiovascular system is of utmost importance in human development, and the related congenital malformations are the result of deviations from a complex dynamic process, occurring during pregnancy [1]. This process is highly regulated by gene expression, and delicate control is mandatory [2]. The use of modern imaging techniques has allowed for a more accurate and earlier diagnosis [3]. On the other hand, the progress of cardiac surgery and catheter intervention has broadened treatment options and improved prognosis [4].

Atrial septal defects (ASDs) are particularly common, comprising around 5–10% of all cardiac malformations [5], with predominance in females (2:1) [6]. Its prevalence in childhood is approximately 3.89‰, decreasing in adulthood to 0.88‰, but these values may be underestimated.

ASDs are characterized by abnormal communication between the left and right atria. According to the location of the defect, four different types of ASDs are considered: *ostium secundum* ASD, *ostium primum* ASD, *sinus venosus* ASD and coronary sinus defect.

Small, significant non-hemodynamic ASDs are usually asymptomatic and may close spontaneously in the first few years of life, but larger defects may have clinical significance either in childhood or later in life. Appropriate and timely approaches are therefore important for therapeutic success.

Deepening our understanding of the mechanisms involved in ASD formation is also fundamental for developing new treatment strategies. To achieve this goal, contributions from different medical fields, such as pathology, cardiology, radiology and genetics, should be gathered and combined.

In this article, we investigate cardiac development and embryology, with a focus on atrial septal defects and their clinical implications.

## 2. Heart Development

The heart develops from the cardiac mesoderm, more specifically from the asymmetrical and bilateral plates, which express NKX2.5 and GATA-4 [7], genes associated with cardiac development. The plates develop and form a tube-shaped primitive heart, with an inner layer—the endocardium—surrounded by the myocardium; the two layers are separated by an extracellular matrix—the cardiac jelly [8]. In this tube phase, inflow occurs in the caudal extremity and outflow in the cranial pole. By the third week, the myocardium acquires contractility. The heart tube undergoes elongation, looping and septation, and in this process, the cardiac valves, conduction system and coronary arteries are formed [9].

### 2.1. Heart Tube Formation and Looping

Early mammalian cardiogenesis occurs in the cranial/cephalic mesoderm. During the presomitic stage, progenitor cells leave the cranial end of the primitive streak and become cardiac progenitor cells, localized on either side of the primitive streak and forming the right and left primary heart fields [10].

By the 19th day after fertilization, a pair of tubes, lined by the endothelium, develops on each side of the midline of the embryonic disk [11]. Due to complex morphogenetic movements of the developing cephalic fold, these bilateral fields of the cardiogenic mesoderm move to the midline and fuse, forming the cardiac crescent [12].

Days later, with the folding of the embryo, the tubes are fused and form the primary heart—shaped as a tube [13]. The mesoderm that involves the tube develops, giving origin to the myocardium, with a layer of the extracellular matrix in-between [14,15].

The second heart field (SHF) originates from the pharyngal mesoderm, from another class of the progenitor cell population, which migrates into the dorsal position of the cardiac crescent. The addition of these cardiac progenitor cells to early tubes contributes to the growth and extension of the developing organ [16]. Neural crest cells communicating with SHF progenitor cells also play an important role in heart tube elongation [17].

By the 22nd day, the heart tube elongates and undergoes rightward looping [18]. This right looping is not a random event but rather an orchestrated phenomenon regulated by genes not yet understood. After this step, there is an activation of multiple genes, in a cascade pattern, which determine the right–left symmetry. The genes involved include *LEFTY*, *NODAL* and *PITX2* [19].

The heart tube bends towards the ventral body and experiences torsion around its central axis, acquiring a configuration of a helix with a counter-clockwise twist (left); it progressively becomes a more complex helix shape with the caudal part exhibiting a left-handed twist and the cranial part a right-handed twist [20]. This looping is crucial, since it promotes proximity between the tube segments, a necessary condition for later establishing the four-chamber connection. The looped tube has a unidirectional blood flow, regulated by the cardiac valves [21].

### 2.2. Development and Septation of Cardiac Chambers

The formation of the future mature heart implies/is driven by a pattern of inductive and inhibitory signals from the adjacent endoderm, ectoderm or midline regions of the embryo in some cases with mutual interactions. The contribution of an intricate and complex set of structural and transcription genes and other signaling molecules, including GATA4, NKX2-5, TBX5, HAND2, ISL1, Sonic Hedgehog or Wnt, highlights the complexity of heart and atrial septum development [22].

The recently looped heart tube experiences ballooning growth, providing shape for the atria and ventricles; the atria arise from the dorsal aspect of this looped tube, while the ventricles arise from the ventral aspect [21]. Atrial growth is bilateral and in parallel, while ventricular growth is unilateral and sequential [23]. The atrial and ventricular myocardia develop muscular trabeculations, and the cardiac jelly is excluded, remaining only in the atrioventricular canal and outflow regions [8]. These cardiac trabeculae are in essence muscular ridges that are present in the heart ventricles. They arise from asymmetric division for the cardiomyocytes, in a process called oriented cell division, which induces the differentiation and directional migration of a proportion of cardiomyocytes in the compact zone [24]. This mechanism is vital; a lack of trabeculation will cause embryonic lethality once the coronary vasculature system starts to function in the embryonic heart due to compromised cardiac function [25].

The jelly is populated with cells derived from the endocardial cells, forming cardiac cushions that facilitate cardiac septum development [26].

The partitioning of the atrioventricular canal during the ballooning process includes the contribution of the atrioventricular endocardial cushions; these cushions arise from the cardiac jelly located at the atrioventricular junction, first from the delamination and transformation of endocardium cells [27]. Some cells undergo the epithelial–mesenchymal transition, migration and proliferation, ultimately leading to the formation of the first cardiac septa with the valves included [28]. The chambers forming the myocardium show specific gene expression, involving *ANF*, *CX40* and *CX43* but excluding *TBX2* and *TBX3*, which are present in the primary heart tube and act as repressors of myocardium development [29]. Interestingly, there are gene expression differences between the left and right ventricles, with the cardiac transcription factor *TBX5* being more expressed in the left ventricle [7]. On the other hand, the myocardial compaction of the outer layers occurs in the absence of *ANF* and *CX40* activation, which implies the existence of different regulation processes [30].

Initially, the atria are connected only to the left ventricular segment, without direct communication with the right ventricle [31]. Nevertheless, blood can reach the right ventricle through the interventricular foramen. This foramen is created because of the differential growth of the right ventricular inlet. The ventricular outlet is initially connected solely to the right ventricle, but again due to ventricular differential growth, it becomes connected to both ventricles [32].

Regarding the ventricular septum, its muscular component grows upward from the apex towards the endocardial cushions. The membranous component is formed by both the downward growth of the aorticopulmonary septum and the posteroinferior proliferation of cells derived from the endocardial cushions [33].

The atria incorporate the draining veins and develop a pair of valves around the *sinus venosus*. The fusion of the anterior part of these valves creates the *septum spurium.* To its left, the primary atrial septum (*septum primum*) begins to form in the roof of the common atria and grows downward [34]. The primordial atrial septum begins with a small ridge of mesenchyme that persists after the retraction of the atrial cardiac jelly [35]. The leading edge of this mesenchymal bridge expresses the transcription factor Tbx3 as it grows from the atrial roof towards the atrioventricular cushions, forming the primary septum [36].

**Figure 1:**
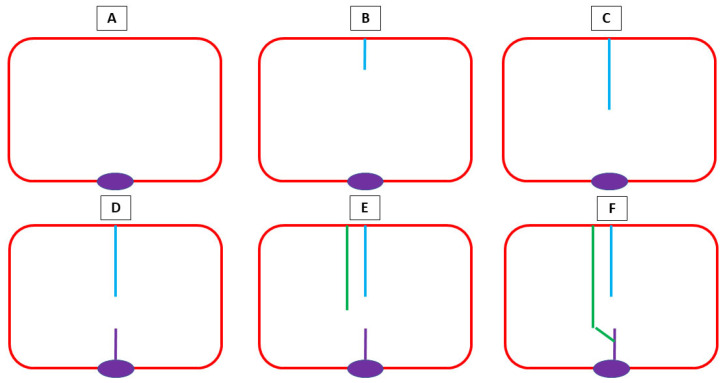
A schematic representation of atrial septation. (**A**) An atrium without septation with an endocardial cushion (purple); (**B**) the *septum primum* (blue) emerges from the superior part, defining an opening between it and the endocardial cushions—the *ostium primum*; (**C**) the *septum primum* elongates; (**D**) on the 37th day within the primary septum, the *ostium secundum* arises, being the space between the upper part (blue) and lowest part (purple) of the *septum primum*; (**E**) the *septum secundum* (green) develops, partially closing the *ostium secundum*, before fusing with the lowest part of the *septum primum* (**F**).

Between the lower border of the *septum primum* and the endocardial cushions, there is an opening—the *ostium primum* [37]. Before the *septum primum* fuses with the endocardial cushions, an opening arises on the 37th day within the primary septum—the *ostium secundum* [34]. This ostium is then partially closed by another enfolding rising from the atrial roof—the *septum secundum* [38]. The inferior extremity of the *septum secundum* then fuses to the lowest part of the *septum primum*. This process is represented in Figure 1.

At the atrioventricular junctions, dorsal and ventral endocardial cushions [39] fuse in the midline, dividing a single atrioventricular canal in the right and left orifices. The fusion of the cushions with the developing interventricular muscular septum and the septum primum completes the septation of the atrial and ventricular chambers [40]. The lower part of the septum primum does not degenerate and acts as a rim/lid/cover, allowing for the right-to-left shunting of oxygenated blood during gestation [37].

Due to the importance of the endocardial cushions, anomalies in their development are related to several cardiac malformations involving septation, such as atrial, ventricular and atrioventricular septal defects, as well as great artery defects [34]. Some studies have related endocardial cushion defects to genetic alterations, such as *VEGF* polymorphisms [41].

The cardiac outflow tracts have an origin that is a little more complex; they derive partially from the primary heart tube and partially from the ingrowth of cells from the neural crest and rim of the secondary cardiac plate. The interaction between these cells originates in the ventricular outflow tracts, the arterial valves and the intrapericardial parts of the aorta and pulmonary trunk [42].

The pericardium, which is a sac-like structure, develops straight around the heart tube.

The epicardium derives from a transient structure called the proepicardial organ—a cluster of mesothelial progenitor cells located at the venous pole of the heart tube and coordinated by the expression of *NKX2.5* and *ISLET1* in precursor cells [43]. These cells migrate to the surface of the looping heart, enveloping the myocardium; some experience the epicardial–mesenchymal transition and infiltrate the myocardium, differentiating into several types of cells, such as fibroblasts and muscular cells; others remain in the surface of the heart and form the epicardium.

The coronary arteries and veins develop by vasculogenesis and angiogenesis mechanisms from cells that grow over the myocardium (derived from the proepicardium) [44].

Finally, the conduction system develops from the myocardium of the primitive heart tube; the fatty and nervous plane between the atrial and ventricle myocardium only occurs after septation is completed (usually at the 7th week) [45].

Atrial septal defects have been shown to have several complex and multifactorial molecular mechanisms. Deepe and colleagues reported that TGFbeta and BMP signaling plays a role in the formation of the mesenchymal cap associated with the primary atrial septum [35]. Among the transcriptional genes associated with atrial septum development, *TBX5* (primary and secondary septum defects), *GATA4* (secondary septum defects), *GATA6* (primary septum defects) and SOX9 (primary septum defects) have been reported/mentioned. In addition, the role of Wnt signaling is known in the development of the primary atrial septum among other heart structures. *NKX2.5* mutation carriers have been described to carry atrial septum defects. *TBX20* mutations have also been related to atrial septum defects mainly due to the synergistic interactions of *TBX20* with *NKX2.5* and *GATA4* [46].

Additionally, factors such as maternal alcohol consumption, smoking, antidepressant drugs and diabetes are also involved in atrial septal defects [47].

## 3. Fetal Blood Circulation and Changes After Birth

### 3.1. Fetal Circulation

In the fetal period, blood is oxygenated via the placenta and returns to the fetus via the umbilical vein that joins the portal vein at the hepatic hilum [48]. The umbilical vein therefore ensures liver metabolic and oxygen supply, but the majority of its blood bypasses the hepatic parenchyma through the ductus venosus to the inferior caval vein and right atrium [49]. The oxygenated blood supplied to the heart reaches the left-sided chambers via the foramen ovale, where the blood is pumped and distributed to the systemic circulation. The majority of this flow is diverted to the head and upper limbs, which require a higher consumption of oxygen; only about ¼ crosses the aortic arch and reaches the descending aorta, where it mixes with the less oxygenated blood coming from the arterial duct [50].

Deoxygenated blood from the upper body returns to the heart via the superior caval vein and then goes preferentially to the right ventricle and pulmonary trunk. However, just a small amount of this flow goes to the lungs due to the high pulmonary resistance of the non-functional parenchyma. The remaining blood transverses the arterial duct to the descending aorta [48] and lower half of the body.

Therefore, contrasting with the adult circulation, there is a mixing of oxygenated and deoxygenated blood at some points of the fetal circulation [50].

### 3.2. Postnatal Circulatory Adaptations

After birth, the fetal lungs assume the function of oxygenating the newborn [51]. The blood from the right ventricle now goes straight to the lungs and not to the arterial duct, which closes around 10–15 h after birth. This leads to an increased pulmonary outflow and consequent rise in the left atrial pressure, which pushes the flap of the oval fossa against the septum and seals the atrial septal orifice.

## 4. Malformations of the Interatrial Septum

Atrial septal defects can occur as isolated lesions in an otherwise normal heart or can be associated with other more complex cardiac congenital defects. They may also appear as a sole septal orifice or as multiple defects, in what is called fenestrated ASDs.

Based on the orifice location within the atrial septum, four different types of ASDs are considered (in order of prevalence): *ostium secundum* ASD, *ostium primum* ASD, *sinus venosus* ASD and coronary sinus ASD.

### 4.1. Ostium Secundum Atrial Septal Defect

An *ostium secundum* atrial septal defect is the most frequent (about 75%) [52] and corresponds to the interatrial communications developed in the oval fossa region. These defects may either be the result of excessive *septum primum* apoptosis and reabsorption or a consequence of the insufficient growth of the *septum secundum*, thus being a late event in atrial septation. It is more prevalent in females than males, with a female-to-male ratio of around 2:1 and is more common in premature newborns and those with Down syndrome. This type of defect can be diagnosed at any age, including early infancy, but it may be clinically unapparent [53]. Genetically, it is usually associated with chromosome 21 mutations, as explored in the next section.

### 4.2. Ostium Primum Atrial Septal Defect

An *ostium primum* atrial septal defect corresponds to the second most common interatrial communication, comprising nearly 20% of cases [52]. In this subgroup, communication persists when the *septum primum* fails to fuse with the endocardial cushions, thus leaving an opening between both atria near the atrial floor, being a mid/late event in atrial septation. Due to the association with endocardial cushion abnormalities, this defect may be associated with either atrioventricular valve anomalies, such as mitral valve cleft, or a complete atrioventricular septal defect. Some authors even consider this type of defect as part of the spectrum of atrioventricular septal defects. Most of the genetic alterations associated with this defect involve chromosome 22, as explored in Section 5.

### 4.3. Sinus Venosus Atrial Septal Defect

The communication involved in a *sinus venosus* atrial septal defect represents about 10% of cases, and this defect is located in the atrial septum near the entrance of the inferior or superior caval vein [52]. The superior caval vein is more commonly involved, and a partial anomalous pulmonary venous return is often associated with this, with one or more pulmonary veins not draining directly into the right atrium.

From an embryological point of view, this defect is the result of an improper incorporation of the *sinus venosus* into the right atrium during cardiac development. The majority of cases are sporadic, but some are inherited and associated with mutations in specific genes such as *NKX2-5* (also associated with atrioventricular conduction defects), *GATA4*, *TBX5* (associated with Holt–Oram syndrome) and *MYH6* (implied in septal defects) [54,55].

### 4.4. Coronary Sinus Atrial Septal Defect

A coronary sinus atrial septal defect is the rarest type and is the result of a partially or completely unroofed coronary sinus [52]. Blood from the left atrium enters in the coronary sinus, through a route independent of the atrial septum, and then drains into the right atrium. A persistent left superior caval vein draining into the coronary sinus is usually present. Due to its rarity, the genetic association is not very well established, but it is usually associated with the already mentioned genes *NKX2-5*, *GATA4*, *TBX5* and *MYH6* [55].

## 5. Etiology

The majority of atrial defects are sporadic, without an inherited underlying pattern; however, rarer familial forms have been described.

In both cases, several genetic factors seem to play a role in the appearance of malformations. Genetic mutations in cardiac transcription factor genes such as *NKX2-5*, *GATA4*, *TBX5* and *MYH6*, located in chromosome 14q12, have been associated with atrial septal defects. Furthermore, first-degree relatives of ASD patients are at a higher risk of having the disease [56,57,58,59]. Several syndromes show a defect of the *ostium secundum* type, such as Holt–Oram syndrome, Ellis van Creveld syndrome, Noonan syndrome, Budd–Chiari syndrome and Jarcho–Levin syndrome. In Holt–Oram syndrome, characterized by a mutation in the *NKX2-5* gene, an interatrial septum defect is found in nearly 2/3 of cases, reinforcing the role of this gene in the pathogenesis of these defects [58].

T-box protein 1 gene, *TBX1*, haploinsufficiency is a condition where only one copy of the gene is present. This gene is coded by chromosome 22 and is involved in the development of several muscles and is associated with atrial septal defects—mostly the osteum primum type (and also ventricular septal defects), as seen in DiGeorge syndrome (22q11 deletion), where the gene deletion contributes to characteristic cardiovascular malformation [60]. This relation between chromosome 21 alterations and atrial septal defects is also evident in Down syndrome, where it is the third most common defect [61,62]. Noonan syndrome is also associated with atrial septal defects, especially in cases with *PTPN11* mutations—mostly the *ostium secundum* type [63,64].

Recently, the transcriptome has been pointed out as being important for atrial septal defect development, thus having a major role in this disease. Non-coding RNAs, both long and short, such as *STX18-AS1*, *HOTAIR*, hsa-miR-19a, hsa-miR-19b and hsa-miR-375, have been linked to the progression of atrial septal defects [65].

Environmental factors should also be considered. Alcoholic fetal syndrome, maternal smoking habits in the first trimester and some antidepressants have been linked to a higher incidence of atrial septal defects. Maternal diabetes and advanced maternal age (>35 years) are also risk factors. In patients with Down syndrome, both *ostium primum* and *ostium secundum* ASDs are particularly prevalent [58].

## 6. Physiopathology

In the majority of patients, an ASD provides a left-to-right blood *shunt* at the atrial level [66]. The size of the defect and the atrial relative pressures (in relation to the complacence of the left and right ventricles) are the main determinants of the amount of blood flow deviated [58]. Small defects usually have no significant impact on hemodynamics. In contrast, larger defects promote a substantial passage of blood to the right atrium and right ventricle, leading to volume overload and the dilation of the right heart. An increased amount of blood is then pumped to the pulmonary circulation, leading to vasoconstriction and progressive pulmonary arterial remodeling, which eventually progresses to pulmonary hypertension. In later phases, the chronic volume and pressure overload may impair right ventricle function, resulting in right-sided heart failure.

Understanding these hemodynamic changes is crucial for managing ASDs and preventing long-term complications by providing early diagnosis and appropriate treatment.

## 7. Clinical Aspects and Treatment

Through this manuscript, we investigated normal cardiac (especially atrial) development, highlighting the mechanisms responsible for atrial septum defects. It is important to address their clinical impact and treatment possibilities, thus reflecting the importance of understanding atrial septum defects.

Small atrial septal defects are generally asymptomatic and therefore do not require treatment [67]. Larger defects, however, can lead to an imbalance between pulmonary and systemic output. When this ratio exceeds 1.5, clinical manifestations or long-term complications may arise.

Increased pulmonary flow and the corresponding reduction in systemic output can cause increased respiratory effort, poor weight gain or more frequent respiratory infections during childhood. However, most cases go unnoticed and are often detected during an occasional echocardiogram performed for a heart murmur or for sport screening.

**Figure 2:**
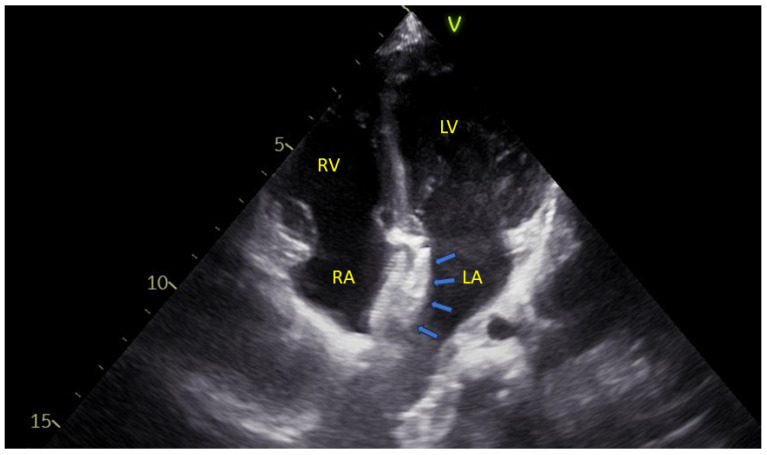
An apical four-chamber echocardiographic view showing the two disks of an atrial septal defect closure device (blue arrows) positioned within the interatrial septum. LA—Left Atrium; LV—Left Ventricle; RA—Right Atrium; RV—Right Ventricle.

An example may be seen in Figure 2.

Most symptoms appear after the fourth decade of life, when the chronically strained right heart starts to show signs of heart failure, such as exercise intolerance, fatigue, lower limb edema and hepatomegaly. At this stage, irreversible pulmonary hypertension may have already developed, which in advanced stages (Eisenmenger Syndrome) will cause a reversal of the interatrial shunt, leading to cyanosis. Palpitations are also common in adulthood and are associated with sinus tachycardia or tachyarrhythmias promoted by the enlargement of the right atrium [58].

Interatrial communication also increases the risk of paradoxical embolism, making stroke a potential initial manifestation of these congenital anomalies.

**Figure 3:**
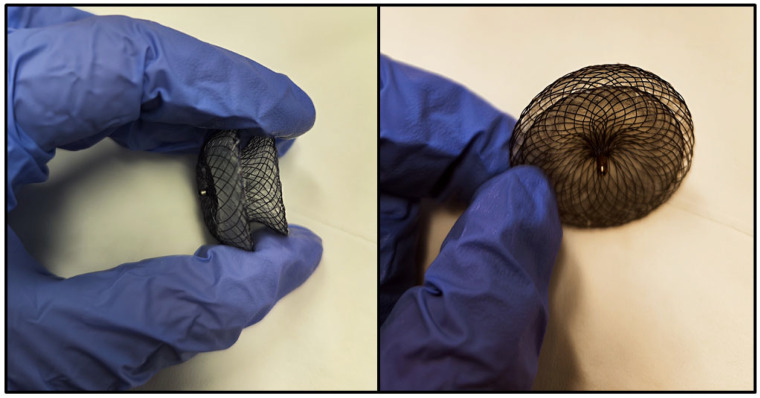
Photographic images of an atrial septal defect closure device, showing its wireframe configuration, consisting of two round disks connected by a narrow waist. Each disk is positioned on opposite sides of the interatrial septum, with the narrow waist traversing the interatrial communication.

Given the natural history of hemodynamically significant atrial septal defects, international guidelines recommend closure either via percutaneous devices (as illustrated in Figure 3) or through surgical means [68,69].

In the choice between these approaches, several factors are considered, such as the type of atrial septal defect (percutaneous closure is indicated only for the secundum type), the size of the opening and the dimension of the interatrial rims [70].

A summary of the several types of ASDs and their features and treatment options can be seen in Table 1.

## 8. Conclusions

The cardiac development process is very complex and highly regulated in order to ensure correct heart formation and, consequently, correct cardiac function. Several genes come into play and are fundamental for this process. Imaging may be a valuable player in identifying these defects, but a deep understanding of the cardiac embryological process and its mechanisms is fundamental for developing new treatment strategies. ASDs, as major defects, need our attention, thus improving medical care quality.

## Figures and Tables

**Table 1:** A summary of the different types of atrial septal defects (ASDs) and their clinical, embryological and genetic features and some treatment options.

Type of ASD	Prevalence	Anatomical Location	Embryological Cause	Genetic Associations/Syndromes	Clinical Features	Treatment Options
**Ostium** **Secundum**	Around 75%	Fossa ovalis (central part of atrial septum)	Excessive resorption of septum primum or underdevelopment of septum secundum	Chromosome 21 (Down syndrome); GATA4, TBX5, NKX2-5; Noonan syndrome (PTPN11); Holt–Oram syndrome	Often asymptomatic in childhood; symptoms may appear in adulthood (e.g., palpitations, stroke, heart failure)	Usually amenable to percutaneous device closure or surgery if significant shunt is present
**Ostium** **Primum**	Around 20%	Lower atrial septum near AV valves	Failure of septum primum to fuse with endocardial cushions	Chromosome 22q11 (DiGeorge syndrome); TBX1 gene; Down syndrome (also common)	May be associated with AV valve defects (e.g., mitral cleft); often diagnosed early due to murmurs or heart failure	Surgical repair required due to involvement of AV valves and location near conduction tissue
**Sinus** **Venosus**	Around 10%	Near entrance of superior (more common) or inferior vena cava	Incomplete incorporation of *sinus venosus* into right atrium	Often sporadic; NKX2-5, TBX5, GATA4, MYH6; Holt–Oram syndrome	Often associated with partial anomalous pulmonary venous return; may remain asymptomatic until adulthood	Surgical repair only (not suitable for percutaneous closure due to location and associated anomalies)
**Coronary Sinus** **Defect**	Rare	Coronary sinus (unroofed or partially unroofed)	Incomplete development or unroofing of coronary sinus wall	Less well understood; associated with NKX2-5, GATA4, TBX5, MYH6	Frequently associated with persistent left superior vena cava; may present with cyanosis or paradoxical embolism	Surgical correction is necessary; anatomy too complex for catheter-based closure

## Data Availability

No new data were created or analyzed in this study.
